# Validation of Volume Calibration by Echocardiography for Invasive Ventricular Pressure Volume Studies in Transcatheter Valve Interventions

**DOI:** 10.1016/j.shj.2024.100307

**Published:** 2024-05-13

**Authors:** Mark M.P. van den Dorpel, Antoon J.M. van den Enden, Sarah Verhemel, Rik Adrichem, Claire B. Ren, Isabella Kardys, Rutger-Jan Nuis, Joost Daemen, Jan Schreuder, Marcel L. Geleijnse, Alexander Hirsch, Nicolas M. Van Mieghem

**Affiliations:** a Department of Cardiology, Cardiovascular Institute, Thoraxcenter, Erasmus University MC, Rotterdam, The Netherlands; b Department of Radiology and Nuclear Medicine, Erasmus MC, Rotterdam, The Netherlands

**Keywords:** Cardiac mechanoenergetics, Echocardiography, Hypertonic saline, Pressure-volume loop, Transcatheter valve intervention, Thermodilution, Volume calibration

Invasive pressure-volume (PV) assessment with a conductance catheter is the gold standard for the real-time evaluation of ventricular cardiomechanics. The conductance catheter relies on Ohm’s conductance-to-volume equation, which translates segmental conductance into a marker of volume. A baseline appraisal of ventricular volume is mandatory to calibrate the conductance catheter signal.

Validated volume calibration involves thermodilution and hypertonic saline boluses (2x 10 mL NaCl 5.0%) in the right ventricle (RV) or left ventricle (LV) to determine the proportionality constant and the so-called parallel conductance created by the surrounding tissues.^1^^,^^2^ Hypertonic saline will augment blood conductivity. Actual ventricular volume is determined by subtracting the parallel conductance fraction from the total conductance.^3^ The proportionality constant is derived from the cardiac output that is obtained by thermodilution. This volume calibration method is invasive, time-consuming, and presents intrinsic measurement errors in the presence of significant tricuspid or mitral regurgitation.

Echocardiography is readily available in catheterization laboratories for ventricular volume assessment and could be used for noninvasive volume calibration.^4^^,^^5^ The main objective of this pilot study was to compare the established volume calibration method by hypertonic saline and thermodilution with noninvasive volume calibration by two-dimensional transthoracic echocardiography (2D-TTE) and three-dimensional transesophageal echocardiography (3D-TEE).

We included all consecutive patients in whom a conductance catheter was introduced during transcatheter valve interventions at the Erasmus University Medical Center, Rotterdam, The Netherlands, between July 2022 and August 2023. Patients with atrial fibrillation or frequent ectopic ventricular beats (defined as ≥7/min) were excluded. All patients provided written informed consent, and the study was conducted according to the Declaration of Helsinki.

A 7F conductance catheter (CD Leycom, Hengelo, The Netherlands) was inserted through femoral arterial or venous access and positioned in the LV or RV apex, respectively. A pulmonary artery catheter was introduced through right jugular venous access for cardiac output measurement by thermodilution and hypertonic saline administration. In patients who underwent tricuspid or mitral transcatheter edge-to-edge repair (t-TEER/m-TEER), RV and LV PV loops were created. In patients with planned transcatheter aortic valve replacement (TAVR), LV PV loops were constructed.

In TEER patients, 3D-volume reconstructions were obtained from TEE-derived 4-chamber views at 60 degrees, with focused recordings of LV and RV using full volume mode. Sector width and depth were adjusted to obtain maximum image quality at the highest possible frame rate, and all studies were electrocardiogram-gated. In TAVR patients, LV apical 2 and 4-chamber long-axis views were recorded by 2D-TTE. Echocardiography images were made with the Philips EPIQ ultrasound system (Philips Healthcare, Andover, Massachusetts). In all patients, echocardiography recordings and PV reconstructions were obtained both immediately before and after valve implantation or valve repair. Echocardiographic data and invasive data were acquired within an interval of minutes, and no medications or intravenous fluids were administered in between. TTE was performed in the left lateral decubitus position to allow optimal acquisition of apical images, while TEE and invasive measurements were performed in supine position.

Dedicated, validated imaging segmentation software (3Mensio Structural Heart, Pie Medical, Maastricht, The Netherlands) was used for segmentation of 3D-TEE images into short-axis and long-axis views. Using a stacked model with 8-mm slices, endocardial borders were semi-automatically traced in short-axis views. Long-axis views were used to verify that ventricular contours were properly traced. Full-cycle imaging allowed for accurate measurement of end-systolic and end-diastolic volumes. Four to six consecutive cardiac cycles were used to obtain full-volume 3D-TEE data. The average of three best-quality consecutive cardiac cycles was used in 2D-TTE.

Different software (Caas Qardia, Pie Medical, Maastricht, The Netherlands) was used for segmentation of 2D-TTE images. Endocardial borders were traced semi-automatically on 2 and 4-chamber images to create a spherical 3D LV-mesh model with end-systolic and end-diastolic LV volumes. Agreement between echocardiography-derived and thermodilution-derived volumes was assessed using Bland-Altman plots and by calculation of intraclass correlation coefficients (ICC, two-way mixed, single measure, consistency). Distributions of variables were tested for normality by using the Kolmogorov-Smirnov test and visual inspection of histograms. Data analysis was performed using SPSS version 25.0 (SPSS, Chicago, United States).

In total, 332 patients underwent TAVR, 49 m-TEER, and 18 t-TEER. A conductance catheter was introduced in 57 TAVR, 28 m-TEER, and 13 t-TEER patients. Two TAVR patients, 6 m-TEER patients, and 1 t-TEER patient were excluded due to arrhythmias. Two-dimensional TTE-derived LV volumes correlated well with volumes obtained by hypertonic saline and thermodilution (ICC 0.92 [95% CI 0.88-0.95], *p* < 0.001). Three-dimensional TEE-derived volumes also showed a good correlation with volumes obtained by hypertonic saline and thermodilution for LV (ICC 0.95 [95% CI 0.92-0.97], *p* < 0.001) and RV (ICC 0.81 [95% CI 0.70-0.89], *p* < 0.001). Mean bias, standard deviations, and limits of agreement (±2 SD, according to Bland and Altman) are illustrated in Figure 1. Two-dimensional TTE displayed a negative mean difference, representing echocardiographic underestimation of invasive volumes, while 3D-TEE displayed positive mean differences, representing overestimation.

**Figure 1:**
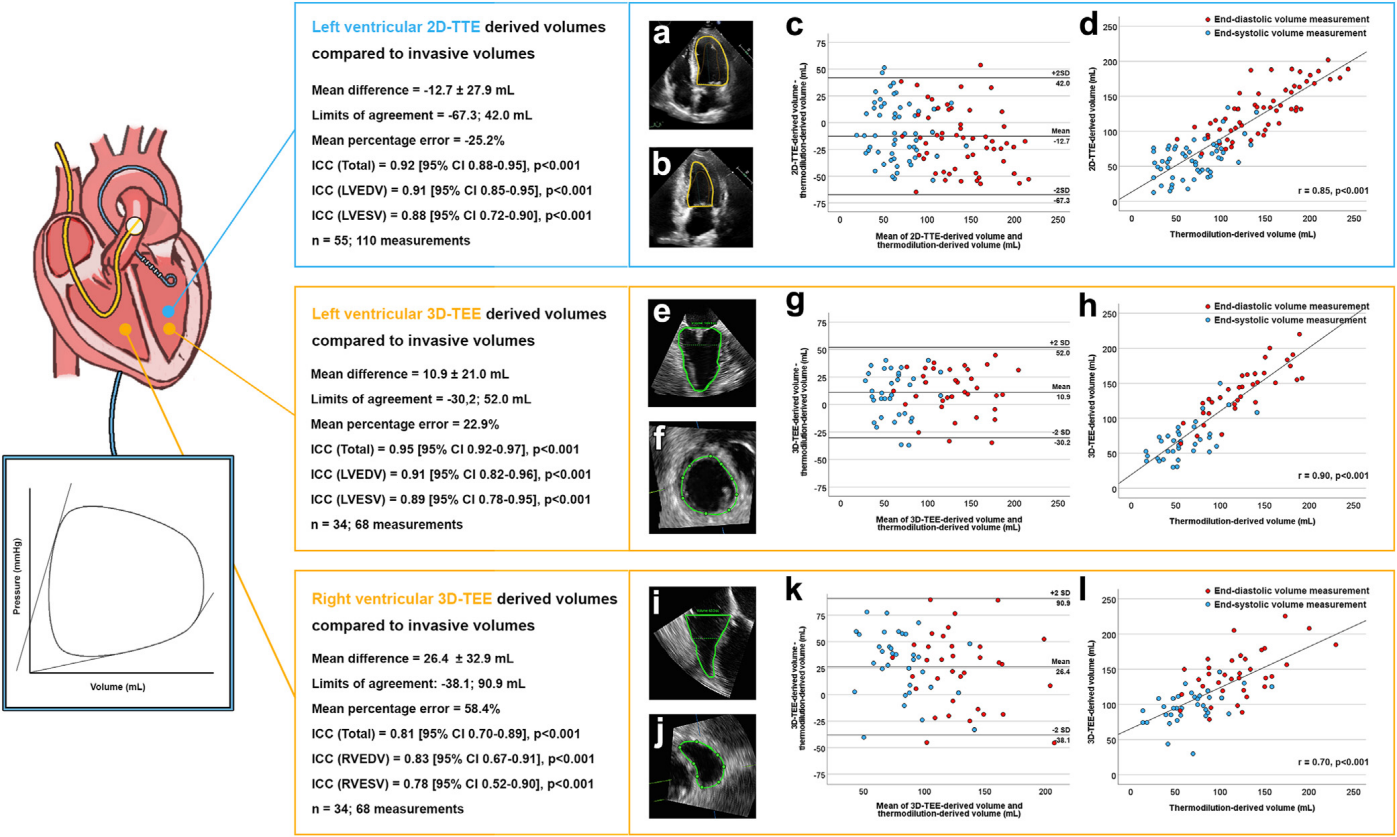
Bland-Altman plots and scatter plots of ventricular volume determined by echocardiography and invasive measurement. (a) 2D-transthoracic echocardiography apical 4 chamber view; (b) 2D-transthoracic echocardiography apical 2 chamber view; (c) Bland-Altman plot showing the mean difference between 2D-echocardiography-derived and invasively measured ventricular volumes; (d) scatter plot of 2D-echocardiography-derived and invasively measured ventricular volumes. (e) 3D-transesophageal echocardiography left ventricular long axis view; (f) 3D-transesophageal echocardiography left ventricular short axis view; (g) Bland-Altman plot showing the mean difference between 3D-echocardiography-derived and invasively measured ventricular volumes; (h) scatter plot of 3D-echocardiography-derived and invasively measured ventricular volumes. (i) 3D-transesophageal echocardiography right ventricular long axis view; (j) 3D-transesophageal echocardiography right ventricular short axis view; (k) Bland-Altman plot showing the mean difference between 3D-echocardiography-derived and invasively measured ventricular volumes; (l) scatter plot of 3D-echocardiography-derived and invasively measured ventricular volumes Abbreviations: 2D-TTE, two-dimensional transthoracic echocardiography; 3D-TEE, three-dimensional transesophageal echocardiography; ICC, intraclass correlation coefficient; LVEDV, left ventricular end diastolic volume; LVESV, left ventricular end systolic volume; RVEDV, right ventricular end diastolic volume; RVESV, right ventricular end systolic volume.

The highest level of agreement, smallest percentage error, and smallest limits of agreements were achieved with LV 3D-TEE, which provided superior image quality and accurate endocardial border tracing. LV 2D-TTE also showed good correlations with invasive measurements. RV 3D-TEE exhibited the lowest level of agreement due to its inherent anatomical (nonspherical) properties, far field, and poorer image quality.

Importantly, we demonstrate strong correlations between invasive and noninvasive volumes but not the equivalence of these measurements. It is plausible that differences between modalities are largely caused by the intrinsic limitations of invasive measurements, due to the presence of significant tricuspid or mitral regurgitation. As such, overestimation by 3D-TEE may in fact reflect an underestimated invasive volume. In TAVR, no significant atrioventricular regurgitation is present, which possibly makes invasive measurements more reliable than in TEER. The underestimation by 2D-TTE in TAVR may be a known limitation of 2D-TTE. Cardiac magnetic resonance imaging would be the best gold standard for validation of echocardiography derived ventricular volume, but this was not feasible in our study due to cost and logistics.

PV reconstructions by invasive and noninvasive volume calibration were retrospectively compared and exhibited similar cardiomechanics, including quantifications of PV area and stroke work (pressure volume area 17,831 mmHg/mL (by invasive volume calibration) vs 17,281 mmHg/mL (noninvasive calibration); *p* = 0.32, and stroke work 11,129 mmHg/mL (invasive) vs 11,520 mmHg/mL (noninvasive); *p* = 0.20 for the total group).

This pilot study shows that, by using selected software for echocardiographic volume quantification, echocardiography can be used as an alternative to invasive hypertonic saline and thermodilution for volume calibration in invasive PV studies. There are various ways to incorporate this into the clinical workflow. Echocardiography images can be transferred to another system for image segmentation and immediate volume calibration in the catheterization laboratory. Alternatively, volume calibration can be performed afterward during PV reconstruction analysis.

## Ethics Statement

The study was approved by the institutional ethics board (reference NL79303.078.22).

## Funding

This research did not receive any specific grant from funding agencies in the public, commercial, or not-for-profit sectors. The study is investigator-initiated and was sponsored by the Erasmus University Medical Center in Rotterdam, The Netherlands.

## Disclosure Statement

N. Van Mieghem received grants or contracts from Abbott, Boston Scientific, Biotronic, Edwards Lifesciences, Medtronic, Pulsecath BV, Abiomed, and Daiichi Sankyo; consulting fees from Jenavalve, Daiichi Sankyo, Abbott, Boston Scientific, and Medtronic; and payment or honoraria for lectures, presentations, speakers, manuscripts, and educational events from Abiomed, Amgen, and Jenavalve. J. Daemen received grants or contracts from Astra Zeneca, Abbott Vascular, Boston Scientific, ACIST Medical, Medtronic, Microport, Pie Medical, and ReCor Medical, and consultancy and speaker fees from Abbott Vascular, Abiomed, ACIST Medical, Boston Scientific, Cardialysis BV, CardiacBooster, Kaminari Medical, ReCor Medical, PulseCath, Pie Medical, Sanofi, Siemens, and Medtronic. A. Hirsch received grants and consultancy fees from GE Healthcare and speaker fees from GE Healthcare and Bayer, and is also a member of the medical advisory board of Medis Medical Imaging Systems. The other authors had no conflicts to declare.

## References

[1] Bastos M.B., Burkhoff D., Maly J. (2020). Invasive left ventricle pressure-volume analysis: overview and practical clinical implications. Eur Heart J.

[2] Chen C.H., Fetics B., Nevo E. (2001). Noninvasive single-beat determination of left ventricular end-systolic elastance in humans. J Am Coll Cardiol.

[3] van den Enden A.J.M., van den Dorpel M.M.P., Bastos M.B. (2022). Invasive real time biventricular pressure-volume loops to monitor dynamic changes in cardiac mechanoenergetics during structural heart interventions: design and rationale of a prospective single-center study. Struct Heart.

[4] Lang R.M., Badano L.P., Mor-Avi V. (2015). Recommendations for cardiac chamber quantification by echocardiography in adults: an update from the American Society of Echocardiography and the European Association of Cardiovascular Imaging. J Am Soc Echocardiogr.

[5] Jacobs L.D., Salgo I.S., Goonewardena S. (2006). Rapid online quantification of left ventricular volume from real-time three-dimensional echocardiographic data. Eur Heart J.

